# A Pediatric Case of Neurodevelopmental Delay with a Familial *H4C11* Variant: Clinical Course and Diagnostic Challenges

**DOI:** 10.3390/jcm15072505

**Published:** 2026-03-25

**Authors:** Elena Tudorache, Andreea Giurgiuveanu, Emilia Severin, Irina-Ioana Iordănescu, Mihaela Anca Bulf

**Affiliations:** 1 Dr. Constantin Gorgos Psychiatry Hospital, 030442 Bucharest, Romania; elena.iordan@drd.umfcd.ro (E.T.); tutuandreea@gmail.com (A.G.); mihaela-anca.dina31@s.fpse.unibuc.ro (M.A.B.); 2 Department of Genetics, Carol Davila University of Medicine and Pharmacy, 020027 Bucharest, Romania; irina-ioana.iordanescu@drd.umfcd.ro; 3 Psychology Major, Faculty of Psychology and Education Sciences, University of Bucharest, 050663 Bucharest, Romania

**Keywords:** Tessadori–Bicknell–van Haaften syndrome 2, *H4C11* gene, intellectual disability, exophthalmos, microcephaly, borderline IQ, case report

## Abstract

**Background:** Tessadori–Bicknell–van Haaften syndrome (OMIM #619759) is a rare autosomal dominant neurodevelopmental disorder associated with heterozygous variants in genes encoding histone H4 proteins. The condition is characterized by global developmental delay, craniofacial dysmorphism, hypotrophy, intellectual disability, and ophthalmologic anomalies. More than 30 individuals with variants in histone H4 genes have been reported to date, reflecting the genetic heterogeneity of this emerging disorder. According to OMIM, the association between the *H4C11* gene and Tessadori–Bicknell–van Haaften syndrome 2 is currently considered provisional. **Methods:** We report the case of a 5-year-old female presenting with expressive language delay, social interaction difficulties, and craniofacial features including microcephaly, exophthalmos, and periorbital fullness (“puffy eyes”). Family history revealed two sisters with borderline intellectual functioning who have not undergone genetic testing. The patient’s father carried the same heterozygous *H4C11* variant (c.97C > T), while maternal testing was negative. **Results**: Neuropsychological evaluation revealed borderline intellectual functioning (IQ 73 at first assessment, 85 at follow-up) with persistent expressive language impairment. Ophthalmologic examination confirmed congenital exophthalmos and hypermetropic astigmatism. Laboratory investigations showed low ferritin and mildly elevated TSH levels, which may have contributed to the observed growth delay. At follow-up, the patient showed an increase in IQ score (73 to 85); however, test–retest variability cannot be excluded. **Conclusions:** This case highlights the importance of careful clinical assessment and cautious interpretation of genetic findings in children with neurodevelopmental delay. Familial segregation of a variant of uncertain significance (VUS), in the absence of functional evidence, should be interpreted conservatively and integrated with detailed phenotypic evaluation to guide clinical management and follow-up.

## 1. Introduction

Tessadori–Bicknell–van Haaften syndrome 2 (TBvH2) (MIM #619759) is a rare autosomal dominant neurodevelopmental disorder characterized by global developmental delay, craniofacial dysmorphism, and variable multisystem involvement. Histones, including histone H4, are essential components of the nucleosome and play a crucial role in chromatin architecture, DNA replication and repair, and transcriptional regulation throughout development. The *H4C11* gene (also known as HISTONE GENE CLUSTER 1, H4 HISTONE FAMILY, MEMBER J or HIST1H4J) (MIM *602826), located within the histone gene cluster on chromosome 6 (6p22.1), encodes a canonical replication-dependent histone H4 [1]. Although canonical H4 proteins encoded by different paralogous genes are identical in amino acid sequence, de novo missense variants affecting highly conserved residues in the H4 globular domain have been implicated in chromatin dysfunction and altered DNA damage response.

In 2017, Tessadori and colleagues first described a neurodevelopmental syndrome linked to variants in histone H4 genes, later designated as Tessadori–Bicknell–van Haaften syndrome 2 (TBvH2). More than 30 individuals with variants in different histone H4 genes have been reported to date. Additional clinical and molecular data are therefore needed to improve understanding of genotype–phenotype correlations and the underlying pathophysiological mechanisms [2].

## 2. Case Presentation: Patient with a Heterozygous *H4C11* Variant

### 2.1. Family History and Early Development

The proband first presented for clinical evaluation at the age of 5 years; genetic testing was subsequently performed at the age of 7 years. The proband was the second child of non-consanguineous parents, with one older and one younger sister. Her family history revealed that both sisters presented borderline intellectual functioning (IQ ≈ 80) and learning difficulties, with unremarkable phenotypic appearance; however, they have not undergone genetic testing.

**Figure 1:**
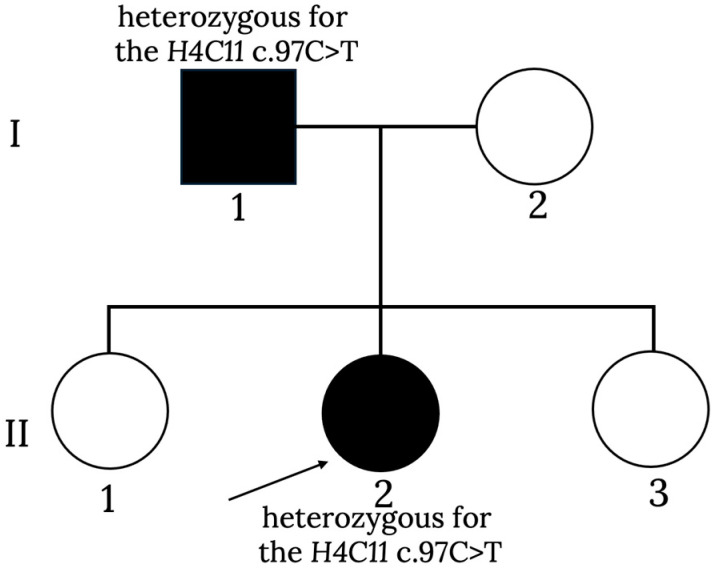
Pedigree of the family. The proband (arrow) and her father carry the same heterozygous *H4C11* c.97C > T (p.Pro33Ser) variant; the mother tested wild-type. The siblings have not undergone genetic testing.

Genetic testing identified the father, aged 42, as carrying the same heterozygous *H4C11* c.97C > T (p.Pro33Ser) variant. He reported a personal history of delayed language development in childhood, with a current IQ of 90 and no major dysmorphic or systemic features. The mother, aged 32, was clinically unaffected, with no relevant medical or genetic findings (Figure 1).

The patient’s personal medical history was unremarkable. The pregnancy was regularly monitored and uneventful, with spontaneous term delivery. Birth weight was 2800 g, and neonatal adaptation was good. However, slow postnatal weight gain was observed, and she was discharged from the maternity hospital after 7 days.

Psychomotor development was appropriate for age milestones: she sat unsupported at 6–7 months, walked independently at 12 months, spoke her first meaningful words at 1 year, and formed short sentences at around 2 years of age.

### 2.2. First Evaluation (Age 5 Years)

At her initial presentation, the parents reported expressive language delay, difficulties in social adaptation, and attention deficits.

**Figure 2:**
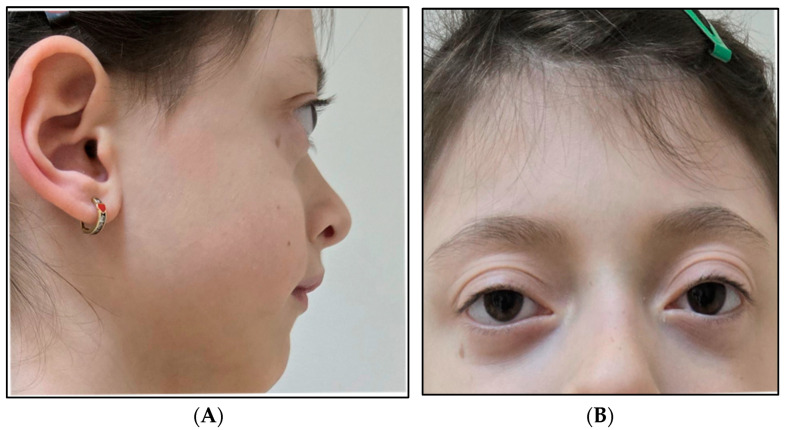
Facial phenotype. (**A**) Lateral view. (**B**) Frontal view. There is noticeable exophthalmos, “puffy eyes” periorbital edema, mild hypertelorism, and a mildly flattened nasal bridge.

**Figure 3:**
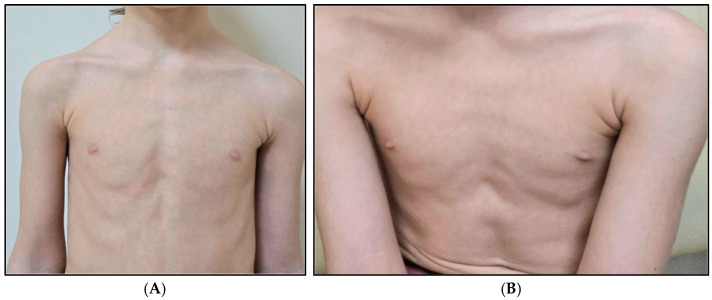
Pectus excavatum (**A**) and kyphotic posture (**B**).

Physical examination revealed a distinctive craniofacial appearance with microcephaly (head circumference: 47 cm, −2 SD for age and sex), exophthalmos with periorbital fullness (“puffy eyes”), mild hypertelorism, a mildly flattened nasal bridge, pectus excavatum, mild kyphotic posture, pale skin, and mild growth delay (weight 17 kg, height 115 cm) (Figure 2 and Figure 3).

Psychological assessment revealed a borderline intellectual level (IQ = 73, Raven Color test), associated with distractibility, impulsivity, behavioral rigidity, and social interaction difficulties.

Laboratory investigations (complete blood count, glucose, urea, creatinine, serum calcium, AST, ALT, TSH, FT4, urinalysis) were within normal limits, except for low ferritin and slightly decreased total serum protein. Relevant laboratory findings are summarized in Table 1.

EEG showed a symmetrical background rhythm without epileptiform discharges.

Based on these findings, the diagnoses were:

- Expressive Language Disorder
- Attention and Activity Disturbance
- Borderline Intellectual Functioning

A behavioral therapy and speech therapy program was initiated, together with neurotrophic supplementation and iron therapy. Further investigations, including ophthalmologic, endocrinological, and genetic evaluations, were recommended.

### 2.3. Second Evaluation (Age 5 Years and 8 Months)

At approximately 8 months after the first evaluation, the patient returned for follow-up. Expressive language delay and social adaptation difficulties persisted, though slight improvements in attention were reported.

Endocrine examination confirmed persistent growth delay (weight 18.5 kg, 12th percentile; height 119 cm, 49th percentile).

Ophthalmologic assessment identified congenital exophthalmos and hypermetropic astigmatism.

Laboratory testing revealed persistently low ferritin, while thyroid function tests remained within the reference range.

Psychological reassessment showed an IQ score of 85 (Raven Color test), corresponding to the borderline range. While the observed increase in IQ score may reflect therapeutic benefit and improved adaptive functioning, test–retest variability cannot be excluded. Expressive language difficulties persisted, both qualitatively and quantitatively.

The patient continued behavioral and speech therapy, and brain MRI was recommended for future evaluation of potential structural anomalies.

Comparative phenotypic analysis with reported OMIM/HPO data is presented in Table 2.

### 2.4. Third Evaluation (Age 7 Years)

Brain magnetic resonance imaging (MRI) performed at the age of 7 years showed no structural abnormalities.

Genetic testing had been performed prior to referral using a commercially available next-generation sequencing (NGS) panel for neurodevelopmental disorders. Detailed information regarding the sequencing platform, coverage metrics, and bioinformatic pipeline was not provided in the original laboratory report.

The analysis identified a heterozygous *H4C11* c.97C > T (p.Pro33Ser) variant, classified as a variant of uncertain significance (VUS) according to ACMG criteria. Variant nomenclature is reported according to HGVS recommendations, using the reference transcript NM_021968.4. According to the laboratory report, the variant is observed at an extremely low frequency in the gnomAD v4.1.0 dataset (total allele frequency < 0.001%).

Parental testing revealed paternal inheritance of the same variant. In silico prediction using the Combined Annotation Dependent Depletion (CADD) algorithm yielded a PHRED-scaled score of 22.9, suggesting a potentially deleterious substitution; however, computational predictions alone are insufficient to establish pathogenicity. The *H4C11* gene is associated with Tessadori–Bicknell–van Haaften syndrome 2 (TBvH2; OMIM #619759), a rare chromatin-related neurodevelopmental disorder. Genotype–phenotype correlations in the present case of Tessadori–Bicknell-van Haaftensyndrome 2 (*H4C11*-related disorder) are presented in Table 3.

### 2.5. Therapeutic Management and Follow-Up

The patient was included in a structured behavioral and speech therapy program, combined with neurotrophic and iron supplementation. After 8 months of follow-up, the patient showed an increase in IQ score from 73 to 85, while expressive language difficulties persisted. Nutritional and endocrine monitoring were continued, and brain MRI was recommended for further etiological clarification. Overall, the patient exhibited a mild neurodevelopmental phenotype within the *H4C11*-related disorder spectrum, with favorable response to therapy, moderate growth delay, and stable general condition during follow-up.

This case description and the accompanying clinical images are published with the written informed consent of the patient’s parents.

## 3. Discussion

Following the clinical description, a review of the literature was undertaken to better define the molecular basis and phenotypic spectrum of *H4C11*-related neurodevelopmental disorders and to highlight the specific features of the present case.

The disorder is characterized by a neurodevelopmental phenotype including global developmental delay, absent or delayed speech, microcephaly, hypotrophy or short stature, and craniofacial dysmorphism such as hypertelorism, abnormal nasal shape, and a wide mouth. Early reports predominantly described more severe clinical presentations [2]. The gene–disease relationship between *H4C11* and TEBIVANED2 is classified as autosomal dominant (AD) in curated databases such as GenCC, with a limited-to-moderate level of evidence and recent updates confirming this association [6]. TEBIVANED comprises multiple subtypes, involving different histone H4 genes, all falling within the same H4-related neurodevelopmental disorder (NDD) spectrum. Subsequent studies have documented expansion of this spectrum, with additional genes and recurrent variants identified [4,5].

Given the paternal inheritance of the variant, the normal-range IQ of the father, and the absence of functional data, no causal relationship between the identified *H4C11* variant and the proband’s phenotype can be established. The findings should therefore be considered hypothesis-generating.

In contrast to the initially described severe forms characterized by profound developmental delay and absent speech, the present patient exhibits borderline intellectual functioning with partial expressive language delay and modest improvement over time (IQ 73→85). This presentation suggests a milder clinical profile compared with early reports, which predominantly described more severe phenotypes [2,4,5]. These observations may indicate that H4-related neurodevelopmental disorders include a broader phenotypic spectrum, ranging from severe intellectual disability to milder neurodevelopmental presentations with borderline cognitive functioning.

The father, who carries the same variant, reported delayed language development in childhood but currently has an IQ within the normal range (~90). This observation highlights the phenotypic variability reported among individuals with histone H4–related neurodevelopmental disorders. However, considering the multifactorial nature of cognitive traits and the current classification of the variant as a variant of uncertain significance (VUS), the phenotypic differences observed between the proband, and her father cannot be attributed solely to the identified variant. The present observation may therefore be compatible with variable expressivity rather than true incomplete penetrance, although the limited number of individuals available for segregation analysis does not allow definitive conclusions.

Additional features observed in the proband, including exophthalmos/periorbital fullness, pectus excavatum, and kyphotic posture, may contribute to refining the clinical description of H4-related neurodevelopmental disorders. However, given the current VUS classification and the absence of functional evidence, these observations should be interpreted cautiously. Nevertheless, the identified *H4C11* variant remains classified as a variant of uncertain significance (VUS), and segregation data alone are insufficient to support pathogenic reclassification currently.

The congenital exophthalmos and hypermetropic astigmatism observed in the present case may represent ophthalmologic manifestations that have been variably reported in earlier cases, including esotropia, oculomotor apraxia, or periorbital edema [4,5]. Iron deficiency was identified in the present patient and may have contributed to the observed growth delay; however, the available follow-up data do not allow a clear assessment of the independent contribution of iron deficiency to the growth trajectory.

Importantly, the heterozygous *H4C11* c.97C > T variant identified in the proband, and her father indicates familial transmission; however, given its current classification as a variant of uncertain significance (VUS), this observation alone does not establish pathogenicity or confirm an autosomal dominant inheritance pattern.

Overall, the present case contributes additional clinical observations relevant to the evolving phenotypic spectrum of *H4C11*-related neurodevelopmental disorders, illustrating a comparatively mild neurodevelopmental presentation. This underscores the importance of comprehensive clinical evaluation in the interpretation of variants of uncertain significance in rare chromatin-related conditions [7,8].

These findings are in line with recent insights into histone-related disorders, as highlighted by Al Ojaimi et al. (2025) [8], who emphasized that defects in histone structure and chromatin remodeling represent an emerging group of epigenetic neurodevelopmental syndromes. Disruption of histone H4 function, such as that caused by *H4C11* variants, can alter nucleosome stability and downstream gene regulation, ultimately leading to widespread transcriptional dysregulation affecting brain development, growth, and morphogenesis. The variable expressivity observed among affected individuals, including our father–daughter pair, reflects the phenotypic heterogeneity typical of this class of disorders, in which chromatin dysfunction can manifest along a continuum from severe intellectual disability to milder cognitive and behavioral involvement. Moreover, as suggested by Al Ojaimi et al. [8], further characterization of histone-related disorders may provide valuable contextual data for variant interpretation and genetic counselling. However, additional functional studies and independent cases are required to clarify the pathogenic mechanisms underlying *H4C11* variation [7].

The present report has several limitations that should be acknowledged. First, this study describes a single clinical case, which limits the ability to draw broader conclusions regarding the phenotypic spectrum of *H4C11*-related disorders. Second, the identified variant remains classified as a variant of uncertain significance (VUS), and no functional studies are currently available to support its pathogenic role. In addition, genetic testing was not performed in the patient’s siblings, which limits segregation analysis within the family. Finally, the genetic testing was performed prior to referral in an external laboratory using a commercially available NGS panel, and detailed information regarding the sequencing platform and bioinformatic pipeline was not available in the original report. Therefore, the findings should be interpreted cautiously and considered hypothesis-generating.

## 4. Conclusions

This case highlights the importance of careful clinical assessment and cautious interpretation of genetic findings in children with neurodevelopmental delay. The presence of a familial variant of uncertain significance (VUS) and a relatively mild phenotype in the affected parent raises the possibility of variable expressivity. Familial segregation of a VUS, in the absence of functional evidence, should therefore be interpreted conservatively and integrated with detailed phenotypic evaluation to guide clinical management and follow-up. Additional clinical reports and functional studies are required to clarify the role of *H4C11* variants in neurodevelopmental disorders.

## Figures and Tables

**Table 1:** Laboratory investigations results.

Parameter	Result	Reference Range	Comment
Thyroid-stimulating hormone (TSH)	2.84 mIU/L	0.670–4.160 mIU/L	Within normal limits
Free thyroxine (FT4)	1.14 pmol/L	0.86–1.40 pmol/L	Within normal limits
Ferritin	10.0 ng/mL	12.8–88.7 ng/mL	Low

**Table 2:** Comparison of clinical features between Tessadori–Bicknell–van Haaften syndrome 2 (OMIM #619759; HPO terms) and the present patient.

Clinical Feature (HPO)	Reported in OMIM/HPO	Present Case	Comments
Global developmental delay	+	Mild developmental delay	Confirmed, improved with therapy
Intellectual disability	+	Borderline IQ (73→85)	Milder phenotype than classical form
Absent/delayed speech	+	Expressive language delay	Partial impairment, responsive to speech therapy
Growth delay/short stature	+	Persistent hypotrophy, moderate growth delay	Weight < P10; mild height delay
Hypotonia	+	Not observed	Normal muscle tone
Muscular atrophy	±	Not observed	Absent in this case
Pes planus	±	Not reported	—
Facial dysmorphism	+	Microcephaly confirmed (head circumference −2 SD), periorbital fullness (“puffy eyes”), exophthalmos, short philtrum	Consistent with syndrome pattern
Hypertelorism	+	Present (mild)	Typical craniofacial trait
Depressed nasal bridge	+	Mildly flattened nasal bridge	Concordant
Downturned corners of mouth	+	Present	Consistent
Wide mouth	+	Mildly wide mouth	Partial expression
Short philtrum	+	Present	Typical
Highly arched eyebrows	+	Present	Consistent
Upslanted palpebral fissures	+	Slight upslant noted	Mild expression
Periorbital fullness/puffy eyes	+	Marked “puffy eyes”, exophthalmos	Characteristic feature
Oculomotor apraxia	±	Not observed	—
Autistic behavior/hyperactivity	+	Hyperactivity, distractibility, social adaptation difficulties	Mild neurobehavioral phenotype
Hypotrophy/muscular thinness	+	Present	Associated with low BMI
Short stature	+	Slightly below average height	—
Hypospadias	± (male only)	Not applicable	—
Endocrine/metabolic alterations	Not consistently reported	Slightly elevated TSH, low ferritin	Possibly contributing to growth delay
Other features	Variable	Pectus excavatum, kyphotic posture	Additional skeletal findings

Legend: “+” = reported feature in OMIM/HPO database; “±” = occasionally reported; “—” = not observed. OMIM reference: #619759—Tessadori–Bicknell–van Haaften syndrome 2 (*H4C11*-related). HPO database consulted: Human Phenotype Ontology, release 2024 [1,3]; Phenotypic features reported in previous studies are summarized based on published data [2,4,5].

**Table 3:** Genotype–phenotype correlation in the present case of Tessadori–Bicknell–van Haaften. syndrome 2 (*H4C11*-related disorder).

Parameter	Description	Comment/Interpretation
Gene	*H4C11* (Histone Cluster 4, H4 family member 11)	Located on chromosome 6p22.1; encodes a canonical histone H4 protein.
OMIM entry	#619759—Tessadori–Bicknell–van Haaften syndrome 2	Rare neurodevelopmental disorder with provisional to moderate evidence.
Variant identified	NM_021968.4:c.97C > T (p.Pro33Ser), heterozygous	Missense variant.
Zygosity	Heterozygous	Variant identified in the proband and her father.
Inheritance pattern	Familial (paternally inherited)	Inheritance pattern cannot be established based on a single family.
Variant classification	Variant of Uncertain Significance (VUS)	Classified according to ACMG criteria.
Method of detection	NGS panel for neurodevelopmental disorders	Testing performed prior to referral using a commercial laboratory.
Father’s genotype/phenotype	Harboring the *H4C11* c.97C > T variant; history of developmental language delay (IQ ~90)	Phenotype differs from that of the proband; IQ within normal range.
Mother’s genotype/phenotype	Wild-type for *H4C11*	Clinically unaffected.
Proband’s phenotype summary	Borderline intellectual functioning, expressive language delay, growth delay, exophthalmos, periorbital fullness, hypotrophy, mild dysmorphism	Clinical features partially overlap with those reported in previous cases.
Segregation evidence	Variant identified in proband and father; mother tested wild-type	Segregation data alone are insufficient to establish pathogenicity.
Population data	Extremely low allele frequency in gnomAD v4.1.0 (<0.001%)	As reported by the testing laboratory.
Variant type	Missense change	Missense change: CADD PHRED score 22.9 suggests potential deleteriousness, although in silico predictions alone are insufficient to establish pathogenicity.

## Data Availability

The data presented in this study are available on reasonable request from the corresponding author. The data are not publicly available due to privacy and ethical restrictions.

## Abbreviations

ACMG	American College of Medical Genetics and Genomics
HPO	Human Phenotype Ontology
IQ	Intelligence quotient
NGS	Next-generation sequencing
OMIM	Online Mendelian Inheritance in Man
SD	Standard deviation
TBvH2/TEBIVANED2	Tessadori–Bicknell–van Haaften syndrome 2
VUS	Variant of uncertain significance
CADD	Combined Annotation Dependent Depletion
